# Participation in Sports Activities before and after the Outbreak of COVID-19: Analysis of Data from the 2020 Korea National Sports Participation Survey

**DOI:** 10.3390/healthcare10010122

**Published:** 2022-01-08

**Authors:** On Lee, Soyoung Park, Yeonsoo Kim, Wi-Young So

**Affiliations:** 1 Korea Institute of Sports Science, Seoul 01794, Korea; fair27@kspo.or.kr (O.L.); thdud0623@snu.ac.kr (S.P.); 2 Department of Physical Education, College of Education, Seoul National University, Seoul 08826, Korea; kys0101@snu.ac.kr; 3 Sport Medicine Major, College of Humanities and Arts, Korea National University of Transportation, Chungju-si 27469, Korea

**Keywords:** COVID-19, Korea National Sports Participation Survey, sports activity, sports participation

## Abstract

The present study aimed to describe the characteristics and rate of participation in sports activities, changes in sports, and the causes of these changes before and after the COVID-19 out-break in Korea using data from the 2020 Korea National Sports Participation Survey (KNSPS). Furthermore, evidence from this study could be used as basic data to maintain and promote sports activities given the current situation, in which the continued spread of infectious diseases, such as COVID-19, is likely. The KNSPS is an annual survey of subjective health and fitness, sports activities and conditions, and participation in sports activities, conducted among a sample comprising the entire Korean population. The current study analyzed data for 9000 participants, and descriptive statistical analysis was performed to calculate the frequency of each item and sample weight. The rate of regular participation in sports activities at least once a week was found to be 60.10% in 2020, representing a decrease of 6.48% from the rate observed in 2019. Among the types of sports facilities frequently used within the residential area, the most common facilities were private sports facilities (22.97%), other sports facilities (20.60%), and public sports facilities (18.97%), although the utilization rate for other sports facilities increased after the COVID-19 outbreak. After the COVID-19 outbreak, 34.12% of men and 29.72% of women responded that there had been a change in their participation in regular sports activities. Both before and after the COVID-19 outbreak, walking was the most common activity, although the participation rate increased from 29.23% in 2019 to 35.70% in 2020. The rankings and participation rates for indoor sports activities (bodybuilding, swimming, etc.) tended to decrease, while those for outdoor sports activities (climbing, cycling, etc.) tended to increase. These changes may be explained in part by the increasing concern regarding infection with increasing age, except among teenagers, and by economic factors. While participation in physical activity provides numerous health benefits, the COVID-19 pandemic has had a negative impact on regular participation in sports activities. The results of this survey suggest that government action is required to enhance participation in sports activities, even in the face of a pandemic.

## 1. Introduction

The first confirmed case of the severe acute respiratory syndrome coronavirus 2 (SARS-CoV-2) infection [1] occurred in Wuhan, China, in December 2019. In March 2020, the World Health Organization (WHO) declared a “pandemic”, the highest level of epidemic alert, due to the rapid worldwide spread of the novel coronavirus disease 2019 (COVID-19) [2]. Variants of SARS-CoV-2 (COVID-19 variants) have continued to emerge since late 2020, resulting in a total of 235,175,106 confirmed cases and 4,806,841 deaths worldwide as of 6 October 2021 [3]. In the Republic of Korea, 321,352 cases of COVID-19 have been confirmed, along with 2524 COVID-19-related deaths [3].

COVID-19 has affected all aspects of politics, economy, society, and culture worldwide, and daily life has changed since the beginning of the COVID-19 outbreak. Infection prevention measures, such as restrictions on travel and social participation, have accelerated the transition to a non-contact society, with many people now staying at home as much as possible. Numerous countries have also implemented social distancing measures, lockdown ordinances, or border blockades in response to the COVID-19 pandemic. In contrast, based on social distancing efforts alone, the Korean government has succeeded in suppressing COVID-19 to a manageable level by focusing on preemptive testing (testing/confirmation), epidemiological investigation using information technology (tracing/epidemiology), and isolation treatment (treatment/quarantine) (i.e., the “test, trace, and treat” (3T) strategy) [4,5]. The willingness of Korean citizens to participate in the voluntary distancing campaign has also been key to the success of the K-quarantine model, which has been highlighted for its success in foreign media [6,7].

However, the implementation of social distancing—which includes various guidelines such as bans on events and competitions with large crowds, self-quarantine of close contacts, restrictions on store business hours, and restrictions on the number of people at private gatherings—has resulted in the commissioning of economic and social activities. Given their economic importance, sports were unavoidable. Most professional leagues in Korea were played without spectators or suspended, and foreign leagues such as Major League Baseball (MLB) [8] and the National Basketball Association (NBA) [9] in the United States postponed the start of their seasons due to COVID-19. Furthermore, an unprecedented situation occurred in which the 2020 Tokyo Olympic Games were postponed. In addition, the main sports facilities used by citizens closed temporarily, which made it impossible for them to participate in sports activities.

Strict measures such as closing leisure and cultural facilities and transitioning to online classes in educational settings have been effective in mitigating the spread of COVID-19. Nonetheless, telecommuting, social distancing, and self-isolation can lead to poor personal health [10]. These measures can place undue strain on an individual’s health by their weakening physical fitness potential, which is positively related to the ability to cope with infection or more serious immunological and cardiopulmonary complications [11,12,13]. In contrast, regular physical activity is associated with both short- and long-term health benefits [14,15] and has been shown to improve mental health by reducing anxiety and depression [16].

In a study of 3800 Spanish adults (age: 18–64 years), self-reported physical activity also decreased significantly after the lockdown, with walking time decreasing by 58.2% and sedentary time increasing by 23.8% [17]. Another study of 3052 Americans reported that symptoms of depression and anxiety were more severe during quarantine/self-isolation than during social distancing. In addition, those who responded that they were active before COVID-19 had a 32.3% decrease in self-reported physical activity after COVID-19, and increased screen time was associated with worsening depression, loneliness, stress, and mental health [18].

A flexible approach is required to improve the general public’s physical, mental, and emotional well-being amid the ongoing COVID-19 pandemic. Such an approach should allow people to participate in sports activities while maintaining adequate social distance and an ability to adapt to individual needs and environmental changes. In other words, one can view the pandemic as an opportunity to expand our approach to athletic participation [14]. The present study aimed to compare the rate of participation in sports activities, changes in sports, and the causes of these changes before and after the COVID-19 outbreak in Korea. Furthermore, evidence from this study could be used as basic data to maintain and promote sports activities given the current situation, in which the continued spread of infectious diseases, such as COVID-19, is likely.

## 2. Methods

### 2.1. Study Design and Procedure

The 2020 Korea National Sports Participation Survey (KNSPS) aimed to identify the actual state of participation in sports activities among Korean citizens based on data provided by national institutions supervised by the Korea Ministry of Culture, Sports, and Tourism. The KNSPS further aims to use these data to develop policies that can promote lifelong participation in sports and maximize satisfaction among citizens. This study used data from the 2020 KNSPS among 9000 Korean citizens aged 10 years or older. The actual period of conducting this survey is from 7 September to 9 November 2020; conducted by a well-trained investigator, respondents were asked to respond by recalling methods “over the past one year period (from 7 September 2019 to 6 September 2020)”. The survey was conducted in the form of one-on-one household visits, and interviews were completed using a structured questionnaire.

### 2.2. Measures

The 2020 KNSPS questionnaire included items related to health status, physical fitness, sports activities and conditions, and the extent of participation in sports activities before and after the COVID-19 outbreak. (a) The rate of regular participation in sports activities was determined based on the number of individuals who regularly participated in sports activities at least once per week in the past year. This rate was determined via a closed-ended question with eight response categories: three or less than three times a month, once a week, twice a week, three times a week, four times a week, five times a week, six times a week, and every day. (b) Utilization of sports facilities in the residential area was assessed using the closed-ended question, “What type of sports facilities do you frequently use?”, for which there were seven response categories: public sports facilities, private sports facilities, other sports facilities, school sports facilities, workplace sports facilities, personal facilities (including home), do not use. (c) Changes in participation in regular sports activities due to the COVID-19 outbreak were assessed via a closed-ended question with yes/no response options: “Did you experience any changes in your regular pattern of participation in sports activities (frequency, sport) compared to before the COVID-19 outbreak (as of March 2020)?” When participants responded yes to item (c), they were asked to report whether they had experienced (d) changes in the frequency of regular sports activities due to the COVID-19 outbreak (i.e., a closed-ended question with five response categories: major decrease, decrease, no change, increase, major increase); (e) changes in the types of regular sports activities (i.e., an open-ended question in which they ranked activities such as walking, basketball, and climbing from first to third in terms of their frequency); (f) and reasons for changes in frequency and/or activity type (i.e., a closed-ended question with seven response categories: concern regarding COVID-19 infection, closure of previously used facilities due to COVID-19, lack of time, health reasons, financial burden, absence of accompanying participants, etc.). (g) Longitudinal (career) participation in specific sports activities was assessed via an open-ended question related to duration, while the reason for pursuing the primary activity was assessed via a closed-ended question with three response options: accessibility, item characteristics, other factors.

Demographic characteristics such as sex (i.e., male/female), age (i.e., categories of 10–19, 20–29, 30–39, 40–49, 50–59, 60–69, and ≥70 years), size of city (i.e., big city: population over 1 million; small city: population over 500,000; town: population less than 500,000), highest educational level achieved (i.e., uneducated, elementary school, middle school, high school, college-less than 4 years, university-at least 4 years, graduate school), and average monthly household income (i.e., categories of <1, 1 to <1.5, 1.5 to <2, 2 to <2.5, 2.5 to <3, 3 to <3.5, 3.5 to <4, 4 to <4.5, 4.5 to <5, 5 to <5.5, 5.5 to <6, and ≥6 million KRW) were also measured.

### 2.3. Statistical Analyses

After defining the population prospects in 2020 based on the survey population included in the Statistics Korea data [19], stratified multi-stage cluster sampling was performed. Weights were applied according to the post-stratification method, and the final sample comprised 9000 people (male = 4503; female = 4497). In cases of inaccurate responses in the data input and verification process, the respondent was directly called to reconfirm the responses. In addition, the data were systematically cleaned and checked in accordance with the guidelines that had been prepared in advance, and errors were re-verified through secondary verification. Descriptive statistical analysis was performed to calculate a simple frequency table and the percentage of each item. All values were rounded to two decimal places.

## 3. Results

### 3.1. Descriptive Statistics

Table 1 shows the demographic characteristics of the participants. After data cleaning, the final number of participants was 9000, and the sex ratio was 1:1. The age group with the highest frequency of participation was the 50–59 years old group (17.99%). Big city (42.63%), high school education (39.19%), and 4–4.5 million won (16.10%) accounted for the greatest proportions of responses for city size, highest level of education, and income, respectively.

### 3.2. Rate of Regular Participation in Sports Activities

Regular participation in sports activities was defined as participation in at least 30 min of exercise once per week over the past year. In Table 2, the rate of regular participation in sports activities was 60.10% in 2020, representing a decrease of 6.48% from 2019 (66.58%). For the first time since the start of the survey in 1986, the rate of regular participation in sports activities was higher among women (60.28%) than men (59.93%). In addition, 29.48% of participants reported that they did not engage in regular sports activities at all, an increase of 3.61% when compared with the rate observed in 2019 (25.87%). When the results were analyzed according to age, rates of participation decreased for all age groups except teenagers when compared with those in 2019, and rates of participation fell sharply for individuals in their 30 s (−11.73%), those with household incomes of 3.0 to 3.5 million won (−16.70%), and those who had attained graduate-level education (−28.10%).

### 3.3. Use of Sports Facilities in Residential Areas

In Table 3, “Private sports facilities” accounted for the highest proportion of facilities used (22.97%), followed by “other sports facilities (20.60%)” and “public sports facilities (18.97%)”. When compared with rates for 2019, utilization rates increased by 6.15% for “other sports facilities”, and 0.50% for “personal facility (including home)”, while they decreased by 3.60%, 2.66%, and 1.51% for “school sports facilities”, “public sports facilities”, and “private sports facilities”, respectively.

Among the types of “other sports facilities”, the most frequently used facilities were public housing complex sports facilities (89.41%), followed by welfare facilities (13.51%) and youth sports facilities (7.47%).

### 3.4. Changes in Sports Activities after the COVID-19 Outbreak

#### 3.4.1. Changes in Regular Participation in Sports Activities due to the COVID-19 Outbreak

In Table 4, among all participants, 34.12% of men and 29.72% of women responded that there had been a change in their participation in sports activities since the COVID-19 outbreak. Among them, 98.12% of men and 96.42% of women responded that it had decreased. When the whole sample was considered, the greatest changes in sports participation were observed for individuals in their 40 s (36.28%), 30 s (34.28%), and 20 s (33.59%). Among those who responded that there had been a change, the frequency of participation decreased most significantly for those in their 30 s (98.24%) and 20 s (98.07%), respectively. When the results were analyzed based on city size, the largest change (34.66%) was observed among individuals living in big cities, with 97.18% of them reporting that their participation had decreased. In terms of final education level, the greatest change (40.88%) was observed among those with university education (4 years or more). Among them, 98.24% of those who reported a change responded that it had decreased. In addition, among those who responded that there had been a change, the frequency of participation in sports activities decreased the most among those with a final education level of elementary school or lower (99.31%) and the least among those with graduate-level education (94.22%). The rate of change in participation in sports activities tended to increase as income increased (18.46–43.12%).

#### 3.4.2. Changes in Regular Participation in Sports Activities

Both before and after the COVID-19 outbreak, the most common activity among Koreans was walking (jogging, trotting), and the rate of participation in walking increased by 6.47% to 35.70% when compared with that before the outbreak (29.23%). After the COVID-19 outbreak, the rankings and participation rates for indoor sports such as bodybuilding (second place 18.07% → third place 8.79%) and swimming (fourth place 10.01% → ninth place 5.07%) tended to decrease. In contrast, the rankings and participation rates for outdoor sports such as cycling and mountain biking (8th place 6.63% → 4th place 8.52%); jumping rope (17th place 2.40% → 11th place 3.86%); and fishing (20th place 1.35% → 14th 2.54%) tended to increase.

#### 3.4.3. Reasons for Changes in Regular Participation in Sports Activities

In Table 5, overall, reported concerns regarding COVID-19 infection tended to increase as age increased, except among teenagers. In particular, concerns were most common among participants in their 60 s (69.35%), followed by those in their 70 s (64.94%). When responses were analyzed according to educational background, facility closure (67.27%) had a greater impact on participation than infection concerns (26.98%) among those with graduate-level education. Except among those with an income of less than 1 million won, the rate of change in participation in sports activities due to concerns regarding infection decreased as income increased (76.10% → 48.94%), while the rate of change due to facility closure increased (21.13% → 48.67%).

## 4. Discussion

In this study, we analyzed data from the 2020 KNSPS to investigate changes in sports participation among Korean citizens before and after the COVID-19 outbreak. In 2020, the rate of participation in sports activities for 30 min or more at least once a week decreased by 6.5% (60.1%) when compared with that 2019. In addition, 34.1% of men and 29.7% of women responded that there had been a change in their participation in sports activities after the outbreak of COVID-19. Among them, 98.2% and 96.4% responded that their participation had decreased, respectively. Similar results have been reported in most countries during the COVID-19 pandemic [20,21,22,23].

Differences in the decrease in participation in sports or physical activity among countries are likely due to differences in the number of confirmed cases and the degree of measures taken to prevent the spread of infection. The results of this survey indicate that the COVID-19 pandemic substantially impacted some characteristics of participation in sports activities among Koreans in 2020. These data may aid in identifying factors that influence participation in sports activities during a pandemic in general.

First, there was a marked decrease in participation in indoor sports activities such as bodybuilding and swimming following the COVID-19 outbreak (Table 6). This may have been due to the closure of public sports facilities and the ban on intermittent gatherings in private sports facilities, which aimed to reduce individual participation and increase social distancing to prevent the spread of COVID-19 (avoiding the 3Cs: “crowded places, close-contact settings, confined and enclosed spaces”).

In addition, this restriction on participation in indoor sports resulted in a relatively larger decrease in overall participation in sports activities among men than among women. This change can be explained by comparing the survey results for 2019 and 2020, which highlight men’s greater preference for bodybuilding and swimming.

The current findings also indicated that spatial restrictions had a significant effect on participation in sports activities. When analyses were performed according to residential area, the frequency of regular participation in sports activities decreased significantly among those living in areas classified as towns. This may be because dependence on public sports facilities is higher in towns and smaller areas than in cities, and the closure of public sports facilities is the number one priority for ensuring social distancing [24].

In addition, utilization rates for other facilities (such as sports facilities in public housing complexes) increased significantly, indicating that citizens wanted to participate in sports activities in a relatively safe space with limited access. Such facilities are advantageous in that they can reduce contact among an unspecified number of people to avoid a high risk of transmission.

Gyms and fitness centers offer a sense of community and personalized training, but they are also crowded places where infections can spread. For this reason, athletic facilities across the United States have been closed during the pandemic to ensure people’s safety [25]. It can be seen that the proportion of “personal facility (including home)” is gradually increasing despite the fact that only 6 months of the investigation period of this study occurred during the period of the COVID-19 pandemic.

Third, from a socio-economic perspective, more substantial changes in sports participation were observed following the COVID-19 outbreak as educational and income levels increased, and the participation frequency decreased overall. In a study of 999 elderly people in Japan, it was found that physical activity decreased by about 5–10% due to COVID-19, and men of a lower socioeconomic status were found to be less physically active (OR = 0.49, 95% CI: 0.30–0.82) [26]. The changes to other sports differed according to the socioeconomic level. Participants with low educational attainment and income tended to transition to sports with good accessibility that did not require additional costs, such as walking and mountain climbing. In contrast, participants with high educational attainment and income tended to transition to sports that could reduce contact with others.

Fourth, multiple factors may explain the decrease in regular sports participation among individuals in their 30 s and 40 s and in the middle-income group. In addition to restrictions on participating in sports commonly enjoyed in this age group (bodybuilding, swimming, etc.), individuals in their 30 s and 40 s are more likely to care for young children, such as preschoolers or elementary school students [27,28]. Due to the COVID-19 pandemic, the demands of childcare have increased due to school restrictions and the implementation of remote classes. Together, these changes may explain the limited participation in sports activities in these groups.

As mentioned above, the COVID-19 pandemic and the restrictions implemented to mitigate its spread have decreased physical activity levels and participation in sports activities worldwide, resulting in negative impacts on global health [29,30]. Overcoming this crisis will involve increasing physical activity and exercise participation, as this will help to enhance immunity and attenuate psychological problems such as stress and anxiety. However, extremely high-intensity or prolonged exercise should be avoided, as this may decrease immune function [31,32,33] and increase the risk of COVID-19 infection or aggravation [34,35,36].

Lately, home fitness products have been gaining increasing popularity because of their convenience and safety. This trend could become the new standard in the near future as strict social distancing rules are enforced due to COVID-19 [25]. In addition, the Internet and related digital platforms, such as smartphone apps, have become essential for education and social interaction [37,38], and these platforms have also shown promise for increasing physical activity among individuals of all ages [39,40,41].

Developing strategies that promote physical activity while mitigating the risk of infection are critical for addressing the social phenomenon introduced by COVID-19. Given the findings of the current study, national efforts are required to support citizens’ choices (preference for outdoor or atypical sports activities, etc.) and remove obstacles (difficulty in raising children, etc.) to public participation in sports activities.

## 5. Conclusions

The results of this study indirectly confirm the changes in sports participation and the influence of socio-economic factors in Korea during the COVID-19 pandemic. At present, when the COVID-19 pandemic will subside remains unpredictable, and social distancing is likely to remain a part of everyday life in the near future. Since sports participation and physical activity are closely related to various health outcomes, the results of this survey suggest that government action is required to enhance the public’s participation in sports activities, even in the face of a pandemic.

In the present era, there is a possibility of future pandemics caused not only by COVID-19 but also by other infectious diseases. Therefore, in future research, as the pandemic continues, it is necessary to follow up on how changes in participation rates in sports, changes in psychological factors resulting from the continuation of such policies as social distancing and the closure of sports facilities, and new types of activities such as home-based exercise and digital platform use will be established.

## Figures and Tables

**Table 1:** Participant characteristics (*n* = 9000).

Variable	*N*	%
Sex		
Male	4503	50.01
Female	4497	49.99
Age category		
10–19	1087	9.92
20–29	1296	14.61
30–39	1301	15.09
40–49	1422	17.38
50–59	1452	17.99
60–69	1268	13.56
70 +	1174	11.45
Size of city		
Big city	3879	42.63
Small city	3041	37.65
Town	2080	19.72
Highest educational level achieved		
≤Elementary school	861	7.31
Middle school	1393	13.44
High school	3496	39.19
College (<4 years)	1386	16.83
University (≥4 years)	1829	22.83
≥Graduate school	35	0.40
Average monthly household income (₩)		
<1 million (USD $1000)	269	2.26
1≤ to <1.5 million (USD $1000–$1500)	380	3.32
1.5≤ to <2 million (USD $1500–$2000)	400	3.62
2≤ to <2.5 million (USD $2000–$2500)	600	5.72
2.5≤ to <3 million (USD $2500–$3000)	651	6.54
3≤ to <3.5 million (USD $3000–$3500)	984	9.66
3.5≤ to <4 million (USD $3500–$4000)	966	9.85
4≤ to <4.5 million (USD $4000–$4500)	1480	16.10
4.5≤ to <5 million (USD $4500–$5000)	1004	11.96
5≤ to <5.5 million (USD $5000–$5500)	936	11.87
5.5≤ to <6 million (USD $5500–$6000)	593	8.31
≤6 million (Over USD $6000)	737	10.79

**Table 2:** Rate of regular participation in sports activities by year (total, unit: %, %*p*).

Variable	Year		Difference (%*p*)
	2020	2019	
Overall	60.10	66.58	−6.48
Sex			
Male	59.93	68.10	−8.17
Female	60.28	65.06	−4.78
Age category			
10–19	52.04	50.06	1.98
20–29	60.94	69.16	−8.22
30–39	58.60	70.33	−11.73
40–49	61.33	70.26	−8.93
50–59	64.38	70.75	−6.37
60–69	62.11	69.01	−6.90
70+	57.04	57.83	−0.79
Size of city			
Big city	61.66	67.22	−5.56
Small city	61.09	66.23	−5.14
Town	54.85	65.87	−11.02
Highest educational level achieved			
≤Elementary school	54.32	55.28	−0.96
Middle school	53.72	53.58	0.14
High school	58.80	66.06	−7.26
College (<4 years)	60.55	67.45	−6.90
University (≥4 years)	67.64	76.42	−8.78
≥Graduate school	58.72	86.82	−28.10
Average monthly household income (₩)			
<1 million (USD $1000)	48.36	58.20	−9.84
1≤ to <1.5 million (USD $1000–$1500)	53.50	56.00	−2.50
1.5≤ to <2 million (USD $1500–$2000)	54.18	58.26	−4.08
2≤ to <2.5 million (USD $2000–$2500)	59.09	65.12	−6.03
2.5≤ to <3 million (USD $2500–$3000)	51.86	59.92	−8.06
3≤ to <3.5 million (USD $3000–$3500)	55.20	71.90	−16.70
3.5≤ to <4 million (USD $3500–$4000)	55.53	66.00	−10.47
4≤ to <4.5 million (USD $4000–$4500)	59.43	64.65	−5.22
4.5≤ to <5 million (USD $4500–$5000)	65.13	66.92	−1.79
5≤ to <5.5 million (USD $5000–$5500)	59.44	68.60	−9.16
5.5≤ to <6 million (USD $5500–$6000)	68.64	71.22	−2.58
≤6 million (Over USD $6000)	70.25	70.90	−0.65

**Table 3:** Use of sports facilities in the residential area by year (total, unit: %, %*p*).

Year	Private Sports Facility	Other Sports Facility	Public Sports Facility	School Sports Facility	Personal Facility (Including Home)	Workplace Sports Facility	Do Not Use
2020	22.97	20.60	18.97	7.61	3.83	0.33	25.69
2019	24.48	14.45	21.63	11.21	3.33	1.11	23.79
Difference (%*p*)	−1.51	6.15	−2.66	−3.60	0.50	−0.78	1.90

**Table 4:** Changes in participation in sports activities due to the COVID-19 outbreak.

Variable	Change in Participation after COVID-19 (%)		Change in Participation Frequency after COVID-19 (%) (Among People Who Responded “Yes” to the Question on the Left)				
	Yes	No	Major Decrease	Decrease	No Change	Increase	Major Increase
Overall	31.92	68.08	25.44	71.89	1.50	1.11	0.06
Sex							
Male	34.12	65.88	23.75	74.37	1.10	0.78	0.00
Female	29.72	70.28	27.38	69.04	1.97	1.48	0.13
Age category							
10–19	27.15	72.85	27.50	70.33	0.79	1.38	0.00
20–29	33.59	66.41	21.37	76.70	1.40	0.53	0.00
30–39	34.28	65.72	25.01	73.23	0.90	0.86	0.00
40–49	36.28	63.72	26.85	69.21	1.38	2.25	0.31
50–59	31.89	68.11	26.33	70.54	1.76	1.37	0.00
60–69	31.41	68.59	24.86	72.41	2.32	0.41	0.00
70+	24.83	75.17	27.28	70.54	1.96	0.22	0.00
Size of city							
Big city	34.66	65.34	21.14	76.04	1.96	0.73	0.13
Small city	32.49	67.51	28.64	69.26	1.06	1.04	0.00
Town	24.90	75.10	30.42	65.98	1.22	2.38	0.00
Highest educational level achieved							
≤Elementary school	22.70	77.30	32.50	66.81	0.30	0.39	0.00
Middle school	23.41	76.59	29.72	67.92	1.67	0.69	0.00
High school	30.38	69.62	24.48	71.82	1.96	1.74	0.00
College (<4 years)	34.14	65.86	21.47	75.85	1.75	0.93	0.00
University (≥4 years)	40.88	59.12	26.10	72.14	0.95	0.60	0.21
≥Graduate school	32.94	67.06	47.17	47.08	0.00	5.75	0.00
Average monthly household income (₩)							
<1 million (USD $1000)	18.46	81.54	25.77	74.23	0.00	0.00	0.00
1≤ to <1.5 million (USD $1000–$1500)	21.71	78.29	39.95	60.05	0.00	0.00	0.00
1.5≤ to <2 million (USD $1500–$2000)	22.25	77.75	21.18	73.46	5.36	0.00	0.00
2≤ to <2.5 million (USD $2000–$2500)	29.93	70.07	25.85	71.07	3.08	0.00	0.00
2.5≤ to <3 million (USD $2500–$3000)	27.49	72.51	19.49	77.82	1.78	0.91	0.00
3≤ to <3.5 million (USD $3000–$3500)	28.03	71.97	27.75	68.87	1.36	2.02	0.00
3.5≤ to <4 million (USD $3500–$4000)	30.59	69.41	26.93	69.46	0.26	3.35	0.00
4≤ to <4.5 million (USD $4000–$4500)	30.66	69.34	24.64	73.59	0.87	0.90	0.00
4.5≤ to <5 million (USD $4500–$5000)	35.21	64.79	27.20	68.23	2.83	1.74	0.00
5≤ to <5.5 million (USD $5000–$5500)	32.18	67.82	25.65	71.28	2.25	0.82	0.00
5.5≤ to <6 million (USD $5500–$6000)	37.61	62.39	22.00	77.02	0.62	0.36	0.00
≤6 million (Over USD $6000)	43.12	56.88	25.15	73.16	0.85	0.42	0.42

**Table 5:** Reasons for the change in regular participation in sports activities due to the COVID-19 outbreak.

Variable	1	2	3	4	5	6	7	8
Overall	55.93	39.75	1.62	1.54	1.07	0.05	0.02	0.02
Sex								
Male	56.36	38.49	1.64	2.35	1.10	0.06	0.00	0.00
Female	55.43	41.19	1.58	0.61	1.05	0.04	0.05	0.05
Age category								
10–19	59.07	33.88	2.35	3.95	0.51	0.24	0.00	0.00
20–29	46.25	49.17	1.12	2.75	0.52	0.19	0.00	0.00
30–39	49.63	48.56	0.53	0.86	0.42	0.00	0.00	0.00
40–49	55.78	40.52	1.09	1.55	1.06	0.00	0.00	0.00
50–59	54.13	39.76	3.95	0.45	1.59	0.00	0.00	0.12
60–69	69.35	26.73	1.06	0.80	2.06	0.00	0.00	0.00
70+	64.94	30.74	1.02	1.73	1.29	0.00	0.28	0.00
Size of city								
Big city	54.11	41.07	2.34	1.13	1.30	0.00	0.00	0.05
Small city	58.83	36.98	0.97	2.22	1.00	0.00	0.00	0.00
Town	54.13	42.67	1.06	1.06	0.60	0.32	0.16	0.00
Highest educational level achieved								
≤Elementary school	54.60	40.52	1.04	2.80	0.65	0.39	0.00	0.00
Middle school	63.82	28.29	2.89	3.88	1.12	0.00	0.00	0.00
High school	62.37	32.01	2.38	1.00	2.18	0.00	0.00	0.06
College (<4 years)	47.55	49.59	1.71	0.50	0.65	0.00	0.00	0.00
University (≥4 years)	50.84	46.88	0.20	1.89	0.00	0.10	0.09	0.00
≥Graduate school	26.98	67.27	5.75	0.00	0.00	0.00	0.00	0.00
Average monthly household income (₩)								
<1 million (USD $1000)	55.58	43.00	0.00	1.42	0.00	0.00	0.00	0.00
1≤ to <1.5 million (USD $1000–$1500)	76.10	21.13	1.26	0.00	1.51	0.00	0.00	0.00
1.5≤ to <2 million (USD $1500–$2000)	71.14	24.38	0.00	4.48	0.00	0.00	0.00	0.00
2≤ to <2.5 million (USD $2000–$2500)	67.44	28.77	0.64	2.51	0.64	0.00	0.00	0.00
2.5≤ to <3 million (USD $2500–$3000)	59.32	36.18	1.79	1.21	0.59	0.52	0.00	0.39
3≤ to <3.5 million (USD $3000–$3500)	51.36	41.61	4.06	0.58	2.39	0.00	0.00	0.00
3.5≤ to <4 million (USD $3500–$4000)	58.36	35.09	3.21	1.95	1.39	0.00	0.00	0.00
4≤ to <4.5 million (USD $4000–$4500)	56.52	40.05	0.98	1.60	0.85	0.00	0.00	0.00
4.5≤ to <5 million (USD $4500–$5000)	53.85	39.97	3.80	1.09	1.10	0.00	0.19	0.00
5≤ to <5.5 million (USD $5000–$5500)	54.76	42.22	0.19	1.93	0.90	0.00	0.00	0.00
5.5≤ to <6 million (USD $5500–$6000)	54.44	41.39	1.08	1.46	1.63	0.00	0.00	0.00
≤6 million (Over USD $6000)	48.94	48.67	0.17	1.43	0.65	0.14	0.00	0.00

1. Worried about COVID-19 infection; 2. Existing facilities closed due to COVID-19; 3. Lack of time; 4. Absence of accompanying participants; 5. Health reasons; 6. Having time to spare; 7. Financial burden; 8. For weight loss.

**Table 6:** Changes in regular participation in sports activities due to the COVID-19 outbreak (1 + 2 + 3 ranking among respondents who have changed their participation patterns after COVID-19 outbreak; top 15 sports, unit: %).

Rank	Before COVID-19 Outbreak	%	After COVID-19 Outbreak	%
1	Walking (jogging, trotting)	29.23	Walking (jogging, trotting)	35.70
2	Bodybuilding (working out at a gym)	18.07	Mountain climbing	15.36
3	Mountain climbing	15.69	Bodybuilding (working out at a gym)	8.79
4	Swimming	10.01	Cycling, mountain biking	8.52
5	Yoga, pilates, tae-bo	9.17	Yoga, pilates, tae-bo	8.12
6	Soccer, futsal	8.69	Soccer, futsal	7.70
7	Golf (including ground and park golf)	7.17	Gymnastics (bare-handed gymnastics, life gymnastics)	7.32
8	Bicycling, cycling, mountain biking	6.63	Golf (including ground and park golf)	5.59
9	Billiards, pool	5.38	Swimming	5.07
10	Badminton	4.44	Badminton	4.13
11	Table tennis	3.58	Jump rope	3.86
12	Gymnastics (bare-handed gymnastics, life gymnastics)	3.58	Billiards, pool	3.73
13	Basketball	3.34	Bowling	2.72
14	Bowling	3.20	Fishing	2.54
15	Aerobics	2.77	Table tennis	2.54

## Data Availability

The data presented in this study are available upon request from the authors. Some variables are restricted to preserve anonymity of the study participants.
